# Diagnostic accuracy of Xpert MTB/RIF Ultra for tuberculous meningitis in HIV-infected adults: a prospective cohort study

**DOI:** 10.1016/S1473-3099(17)30474-7

**Published:** 2018-01

**Authors:** Nathan C Bahr, Edwin Nuwagira, Emily E Evans, Fiona V Cresswell, Philip V Bystrom, Adolf Byamukama, Sarah C Bridge, Ananta S Bangdiwala, David B Meya, Claudia M Denkinger, Conrad Muzoora, David R Boulware, ASTRO-CM Trial Team, Darlisha A Williams, Kabanda Taseera, Dan Nyehangane, Mugisha Ivan, Patrick Orikiriza, Joshua Rhein, Kathy Huppler Hullsiek, Abdu Musubire, Katelyn Pastick, Pamela Nabeta, James Mwesigye, Radha Rajasingham

**Affiliations:** a Division of Infectious Diseases and International Medicine, Department of Medicine, University of Minnesota, Minneapolis, MN, USA; b Division of Infectious Diseases, Department of Medicine, University of Kansas, Kansas City, MO, USA; c Mbarara University of Science and Technology, Mbarara, Uganda; d Infectious Disease Institute, Makerere University, Kampala, Uganda; e Department of Infectious and Tropical Diseases, London School of Hygiene & Tropical Medicine, London, UK; f Foundation for Innovative New Diagnostics, Geneva, Switzerland

## Abstract

**Background:**

WHO recommends Xpert MTB/RIF as initial diagnostic testing for tuberculous meningitis. However, diagnosis remains difficult, with Xpert sensitivity of about 50–70% and culture sensitivity of about 60%. We evaluated the diagnostic performance of the new Xpert MTB/RIF Ultra (Xpert Ultra) for tuberculous meningitis.

**Methods:**

We prospectively obtained diagnostic cerebrospinal fluid (CSF) specimens during screening for a trial on the treatment of HIV-associated cryptococcal meningitis in Mbarara, Uganda. HIV-infected adults with suspected meningitis (eg, headache, nuchal rigidity, altered mental status) were screened consecutively at Mbarara Regional Referral Hospital. We centrifuged CSF, resuspended the pellet in 2 mL of CSF, and tested 0·5 mL with mycobacteria growth indicator tube culture, 1 mL with Xpert, and cryopreserved 0·5 mL, later tested with Xpert Ultra. We assessed diagnostic performance against uniform clinical case definition or a composite reference standard of any positive CSF tuberculous test.

**Findings:**

From Feb 27, 2015, to Nov 7, 2016, we prospectively evaluated 129 HIV-infected adults with suspected meningitis for tuberculosis. 23 participants were classified as probable or definite tuberculous meningitis by uniform case definition, excluding Xpert Ultra results. Xpert Ultra sensitivity was 70% (95% CI 47–87; 16 of 23 cases) for probable or definite tuberculous meningitis compared with 43% (23–66; 10/23) for Xpert and 43% (23–66; 10/23) for culture. With composite standard, we detected tuberculous meningitis in 22 (17%) of 129 participants. Xpert Ultra had 95% sensitivity (95% CI 77–99; 21 of 22 cases) for tuberculous meningitis, which was higher than either Xpert (45% [24–68]; 10/22; p=0·0010) or culture (45% [24–68]; 10/22; p=0·0034). Of 21 participants positive by Xpert Ultra, 13 were positive by culture, Xpert, or both, and eight were only positive by Xpert Ultra. Of those eight, three were categorised as probable tuberculous meningitis, three as possible tuberculous meningitis, and two as not tuberculous meningitis. Testing 6 mL or more of CSF was associated with more frequent detection of tuberculosis than with less than 6 mL (26% *vs* 7%; p=0·014).

**Interpretation:**

Xpert Ultra detected significantly more tuberculous meningitis than did either Xpert or culture. WHO now recommends the use of Xpert Ultra as the initial diagnostic test for suspected tuberculous meningitis.

**Funding:**

National Institute of Neurologic Diseases and Stroke, Fogarty International Center, National Institute of Allergy and Infectious Disease, UK Medical Research Council/DfID/Wellcome Trust Global Health Trials, Doris Duke Charitable Foundation.

## Introduction

Tuberculous meningitis is the second most common cause of adult meningitis in Africa.^1^, ^2^, ^3^, ^4^ Meningitis from tuberculosis leads to fatality in more than 50% of cases, in large part due to difficulty and delay in diagnosis.^5^ Cerebrospinal fluid (CSF) smear microscopy for acid-fast bacilli has poor sensitivity (≤15%) in routine care.^5^ Although mycobacterial culture has higher sensitivity (50–60%), culture is too slow to be clinically useful.^5^

In 2013, WHO endorsed the Xpert MTB/RIF assay (Cepheid, Sunnyvale, CA, USA) as the preferred initial test to investigate tuberculous meningitis after a systematic review of 13 studies.^6^, ^7^, ^8^ The Xpert is cartridge-based fully-automated PCR test. Of three large cohorts, the first reported 67% sensitivity using Xpert in microbiologically proven tuberculous meningitis in HIV-infected South Africans.^9^ This study initially tested 1 mL of CSF but later found higher sensitivity (82%; 22 of 27 positive cases) when centrifuging 3 mL of CSF.^9^ Sensitivity compared with consensus clinical case definition was only 36%.^9^, ^10^ The second large cohort study, in Vietnam, measured Xpert against the same clinical case definition and found 59% sensitivity generally using 2 mL or less of centrifuged CSF.^11^, ^12^ A third Ugandan study reported 28% sensitivity with 2 mL of uncentrifuged CSF and 72% sensitivity when centrifuging a median volume of 6 mL (IQR 4–10) with Xpert.^12^ Thus, the imperfect sensitivity has meant no test can exclude tuberculous meningitis.^13^, ^14^

The Xpert MTB/RIF Ultra (Xpert Ultra) is a new, fully automated, nested real-time PCR assay for the GeneXpert platform. The re-engineered Xpert Ultra has sought to improve the analytical sensitivity for *Mycobacterium tuberculosis* detection and to improve rifampin resistance detection.^15^ We analysed the diagnostic performance of Xpert Ultra for detection of *M tuberculosis* in CSF as compared with Xpert or culture. We hypothesised that Xpert Ultra would have improved sensitivity. Preliminary results of Xpert Ultra performance were summarised in a WHO report in March, 2017.^16^

**Research in context**

**Evidence before this study**

The Xpert MTB/RIF (Xpert) Ultra is a new assay without prior publications. We searched PubMed with the terms ((tuberculous meningitis) or TB meningitis) AND (Xpert MTB/RIF Ultra) for articles published in English up to June 19, 2017, which yielded no results. We also searched Google Scholar for articles related to Xpert Ultra, which yielded one conference abstract, which had been cited by 13 publications relating to the future of tuberculosis diagnostics but without including any data on Xpert Ultra. When we searched PubMed with the terms (Tuberculous meningitis) or TB meningitis) AND (Xpert MTB/RIF), we found and reviewed 21 publications. These publications reported imperfect sensitivity of cerebrospinal fluid Xpert, ranging from 50% to 72% against cerebrospinal fluid *Mycobacterium tuberculosis* culture. A meta-analysis reported that Xpert has a pooled sensitivity of 80·5% against culture and 62·8% against a clinical reference standard. Three opinion pieces point out that with imperfect sensitivity, the Xpert assay cannot be used as a rule-out test for tuberculous meningitis and an assay with improved diagnostic performance is urgently needed.

**Added value of this study**

To our knowledge, we present the first evaluation of the diagnostic performance of Xpert Ultra in the diagnosis of tuberculous meningitis. Xpert Ultra has been re-engineered to improve diagnostic performance with a lower analytic limit of detection, comparable to mycobacterial culture. We assessed diagnostic performance in two ways. First, against any positive cerebrospinal fluid tuberculosis test being defined as definite tuberculosis, and second, against a consensus clinical case definition not including the Xpert Ultra result. Xpert Ultra had higher sensitivity of 95% than either Xpert (45%) or culture (45%) for definite tuberculous meningitis. Based on the consensus clinical case definition, Xpert Ultra found 70% sensitivity for probable or definite tuberculous meningitis. Xpert or culture each had 43% sensitivity for probable or definite tuberculosis based on the clinical case definition (which excluded Xpert Ultra results).

**Implications of all the available evidence**

A diagnostic test exhibiting more than 90% sensitivity for tuberculous meningitis with results available in fewer than 90 min holds great potential to improve patient outcomes, particularly to detect those with the lowest bacillary load. The negative predictive value of 99% against definite tuberculous meningitis is a major step forward in tuberculous meningitis diagnostics. However, negative predictive value was only 93% against probable or definite tuberculous meningitis, and thus clinical judgment remains paramount. Xpert Ultra's combination of lower analytic limit of detection and ability to detect dead bacilli after starting tuberculosis therapy vastly improves sensitivity compared with either culture or Xpert. On the basis of our findings, we believe Xpert Ultra should be a first-line test for tuberculous meningitis after excluding cryptococcal infection. The assay must now be evaluated prospectively in a larger study including in HIV-negative individuals and children, and in which post-mortem evidence of tuberculosis can be included in a reference standard.

## Methods

### Study design and participants

We prospectively obtained diagnostic CSF specimens during screening for the Adjunctive Sertraline for the Treatment of HIV-associated Cryptococcal Meningitis trial (NCT01802385). HIV-infected adults with suspected meningitis (eg, headache, nuchal rigidity, altered mental status) were screened consecutively at Mbarara Regional Referral Hospital in Mbarara, Uganda. Clinical history, general examination, and detailed neurological findings were prospectively recorded, in agreement with the data considered important by Marais and colleagues^17^ for tuberculous meningitis studies. Further investigations, including chest radiography, abdominal ultrasonography, and sputum Xpert MTB/RIF, were undertaken as clinically indicated. Brain imaging was unavailable. Processing of CSF samples is described in the appendix (p 7). Eligible participants were at least 18 years of age with suspected meningitis. All participants or their surrogates provided written informed consent, including for additional future diagnostic testing. Institutional review board approvals occurred.

### Procedures

After lumbar puncture, physicians did cryptococcal antigen lateral flow assay (IMMY, Norman, OK, USA) testing at the bedside. All participants without cryptococcosis were then evaluated for tuberculous and bacterial meningitis; this stepwise diagnostic approach was a deliberate cost-efficient approach.^1^ Participants with cryptococcal meningitis with concern of tuberculous meningitis co-infection were included at physician discretion.

The algorithm for CSF diagnostic testing is fully described in the appendix (p 7). After bedside testing, approximately 1 mL of CSF was removed for routine testing (eg, white cell count, protein, glucose, Gram stain, Ziehl-Neelsen stain), and we centrifuged the remaining volume at 3000 × g for 20 min. We removed and cryopreserved all supernatant except for 2 mL, which we resuspended via vortexing for 15–20 s. We then used 1 mL for Xpert testing, 0·5 mL for tuberculous culture, and 0·5 mL for storage at −80°C awaiting Xpert Ultra availability. Xpert testing was done with 1 mL sample reagent. We did cultures using mycobacteria growth indicator tube (MGIT) with a Bactec960 instrument (Becton Dickinson, Franklin Lakes, NJ, USA) at the Médecins Sans Frontières Epicentre Mbarara laboratory. When Xpert Ultra became available, 0·5 mL of cryopreserved CSF was thawed, 1·5 mL of sample reagent added, and the mixture immediately pipetted into the Xpert Ultra cartridge and run. All CSF testing was prospective except for Xpert Ultra, which we did on cryopreserved samples, albeit planned prospectively. Positive values for all tests were per manufacturer protocols. Clinical data and other test results were unavailable to those performing each separate tuberculous test.

We assessed diagnostic performance of Xpert Ultra in two ways. First, we used the uniform clinical case definition as described by Marais and colleagues.^10^ We categorised participants as definite, probable, possible, and not tuberculous meningitis. We defined definite tuberculous meningitis in accordance with the consensus definition as any CSF test positivity by microscopy, culture, or commercialised PCR (including Xpert or Xpert Ultra).^10^ The definition for definite tuberculous meningitis included PCR testing because culture is an insensitive tuberculous meningitis reference standard,^10^ and we judged the likelihood of tuberculous DNA detection in the CSF of an HIV-infected person with aseptic meningitis representing a false positive result as low (eg, *M tuberculosis* colonisation in the CSF is extremely unlikely and, given the clinical context, a rare lab contamination is less likely than a PCR test indicating true tuberculous meningitis).^7^ We included the fully automated PCR Xpert Ultra test in this composite reference standard with the recognition that, in vitro, its analytical sensitivity is ten times greater than that of Xpert.^15^ This inclusion was prespecified but risks an incorporation bias. Thus, we did a second analysis that did not include Xpert Ultra results as part of the Marais reference standard.^10^

We did additional molecular testing to exclude alternative aetiologies and confirm Xpert Ultra results. First, we retrospectively ran CSF specimens positive only for Xpert Ultra on the FilmArray Meningitis/Encephalitis Panel (BioFire Diagnostics, Salt Lake City, UT, USA), which uses a multiplex PCR to detect nucleic acids of 14 meningitis pathogens of *Streptococcus pneumoniae, Neisseria meningitidis, Listeria monocytogenes, Haemophilus influenzae, Streptococcus agalactiae, Escherichia coli*, herpes simplex virus types 1 and 2, cytomegalovirus, varicella zoster virus, human herpes virus 6, enterovirus, human parechovirus, and *Cryptococcus neoformans/gattii*.^18^

Second, we used next-generation sequencing to verify the presence of *M tuberculosis* DNA by an alternative method. For CSF specimens positive for Xpert Ultra, we extracted the residual volume of liquid left in the Xpert Ultra cartridges, which potentially contained DNA amplicons, and deep sequenced it on an Illumina MiniSeq platform (Illumina, San Diego, CA, USA).^19^ DNA sequencing methodology is further described in the appendix (p 5).

### Statistical analysis

We calculated sensitivity, specificity, negative predictive value, and positive predictive value by defining true or false positives and true or false negatives against the reference standard with inclusion or with exclusion of Xpert Ultra results. We used SPSS version 24 (IBM, Armonk, NY, USA) to compare baseline clinical characteristics and demographic data by diagnosis via Mann-Whitney *U* for continuous variables and Fisher's exact test for categorical variables. We evaluated concordance between diagnostic assays with McNemar's test. We counted invalid tests (eg, culture contamination, Xpert error) as negative results.

### Role of the funding source

The funders had no role in study design, data collection, data analysis, data interpretation, or writing of the report. The corresponding author had full access to all the data in the study and had final responsibility for the decision to submit for publication.

## Results

**Figure 1:**
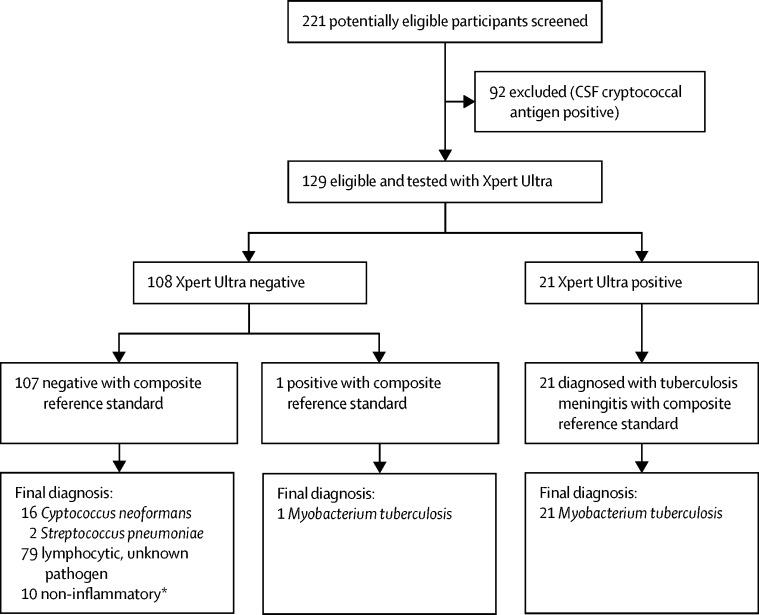
Trial profile Composite reference standard was culture, Xpert, or Xpert Ultra. CSF=cerebrospinal fluid. Xpert=Xpert MTB/RIF. *Suspected meningitis with normal cerebrospinal fluid profile of <5 leucocytes per μL and protein <40 mg/dL.

**Figure 2:**
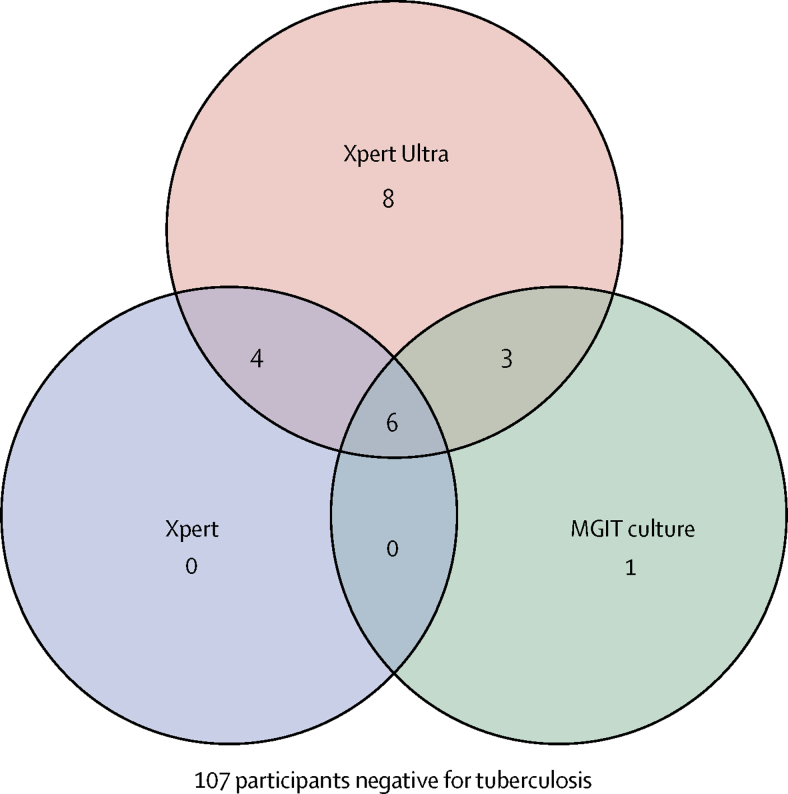
Venn diagram of overlap in tuberculous meningitis diagnostics The Venn diagram displays 22 participants with tuberculosis detected in CSF by each diagnostic test and the overlap between tests. Of eight participants positive by Xpert Ultra only, six had recently initiated HIV therapy, and all eight were negative by testing with cryptococcal antigen, India ink, Gram stain, culture, and multiplex PCR for 14 common meningitis pathogens. Xpert=Xpert MTB/RIF. MGIT=mycobacteria growth indicator tube. CSF=cerebrospinal fluid.

Of 221 HIV-infected people presenting with suspected meningitis between Feb 27, 2015, and Nov 7, 2016, 129 participants underwent tuberculous testing with Xpert, culture, and Xpert Ultra on centrifuged CSF (figure 1). Of these, 113 had a negative CSF cryptococcal antigen and 16 had a positive cryptococcal antigen but had tuberculous meningitis testing at physician discretion out of clinical suspicion. A median of 8 mL (IQR 5–11) of CSF was collected and centrifuged. We diagnosed definite tuberculous meningitis in 22 (17%) of 129 participants tested per the prespecified composite reference standard of any microbiological CSF test positivity (n=10 Xpert; n=10 culture; n=21 Xpert Ultra; figure 2). Participant characteristics of those with and without tuberculous meningitis did not differ aside from lower CSF glucose, higher CSF protein, and a higher proportion with prior tuberculosis among those diagnosed with tuberculous meningitis (table 1).

**Table 1:** Participant characteristics

	**Tuberculous meningitis, composite definition (n=22)**	**Other meningitis (n=107)**	**p value**
Age, years	32 (30–34)	34 (29–43)	0·24
Men	13 (59%)	58 (54%)	0·82
Women	9 (41%)	49 (46%)	0·82
Headache duration, days	7·0 (6·5–14·0)	4·5 (7·0–14·0)	0·83
CD4 T-cell count, cells per μL	72 (43–124)	73 (16–214)	0·92
Serum C-reactive protein, mg/L	49 (11–78)	49 (11–108)	0·86
CSF white blood cells per μL	12 (3–140)	4 (2–15)	0·12
CSF lymphocytes, %	70% (65–85)	70% (62–79)	0·32
CSF total protein, mg/dL	300 (100–565)	170 (53–355)	0·014
CSF glucose, mg/dL	44 (27–65)	69 (49–93)	0·0020
Prior tuberculosis diagnosis	4 (18%)	3 (3%)	0·016
Physician tuberculous meningitis diagnosis*	13 (59%)	45 (42%)	0·24
Alive at discharge or last contact	11 (50%)	75/103 (73%)†	0·045

Data are median (IQR) or n (%). Tuberculous meningitis is defined by composite definition including any positive PCR or culture result. CSF=cerebrospinal fluid.

* Final hospital diagnosis without incorporating any delayed culture results, owing to incubation of up to 8 weeks.

† Four patients left against medical advice, with unknown outcome.

One participant with definite tuberculous meningitis was positive by culture and negative by Xpert and Xpert Ultra (table 2). Of the eight cases detected only by Xpert Ultra, one was receiving antituberculous therapy and six were receiving HIV therapy. The eight cases were also negative for all 14 pathogens on the FilmArray Meningitis/Encephalitis Panel and cryptococcal antigen. Overall, 11 of 22 participants with tuberculous meningitis died during hospitalisation, two were known to have died after discharge, and one was lost to follow-up (table 2).

**Table 2:** Characteristics of participants with tuberculous meningitis

	**Age (years)**	**Sex**	**Headache (days)**	**GCS**	**MRC**	**CD4 (cells per μL)**	**Serum CRP (mg/L)**	**CSF volume (mL)**	CSF white cells per μL*	Lymphocytes*†	Protein*(mg/dL)	Glucose*(mg/dL)	**Xpert**	**Xpert Ultra**	**Culture**	**ART**	**Tuberculous meningitis final diagnosis**‡	**Clinical case status**§	**Alive at hospital discharge**
5004	34	Female	0	15	1	133	2·8	13	··	··	650	28	Positive	Positive	Positive	Yes	Yes	Definite	Yes
5005	34	Male	7	10	3	··	··	12	750	70%	510	23	Negative	Positive	Positive	Yes	No	Definite	No
5103	30	Female	5	14	2	212	7·7	14	120	78%	490	44	Negative	Positive	Positive	Yes	No	Definite	No
5109	22	Female	7	9	3	73	55·4	15	12	60%	50	32	Positive	Positive	Positive	Yes	Yes	Definite	No
5120	36	Female	7	10	3	102	74·1	11	290	90%	260	50	Negative	Negative	Positive	Prior	No	Definite	No
5130	32	Male	14	14	2	67	78·6	10	210	80%	600	49	Negative	Positive	Positive	Yes	No	Definite	No
5162	30	Male	7	15	1	124	7·8	8	610	90%	200	75	Positive	Positive	Negative	No	Yes	Definite	Yes
5170	30	Male	14	13	2	72	64·1	12	5	65%	300	60	Positive	Positive	Negative	Yes	Yes	Definite	Yes
5194	31	Male	7	13	2	249	27·7	14	3	··	250	24	Positive	Positive	Positive	No	Yes	Definite	Yes
5203	49	Male	14	14	2	61	144·0	6	Traumatic¶	90%	410	<20	Positive	Positive	Positive	Yes	Yes	Definite	Yes
5204	34	Male	14	13	2	48	47.4	10	Traumatic¶	··	720	26	Positive	Positive	Positive	No	Yes	Definite	No
5229	32	Male	7	10	3	131	··	5	22	65%	250	60	Positive	Positive	Positive	No	Yes	Definite	No
5233	33	Female	11	14	2	19	19·6	3	140	85%	800	80	Positive	Positive	Negative	No	Yes	Definite	Yes
5285	32	Male	21	14	2	3	147·1	3	1	··	17	71	Positive	Positive	Negative	No	Yes	Definite	Yes
5001	31	Female	5	<15	2	43	264·8	10	0	··	90	17	Negative	Positive	Negative	No	No	Not	No
5269	35	Female	3	14	2	49	8·3	8	2	··	75	110	Negative	Positive	Negative	Yes	No	Not	Yes||
5112	31	Female	14	15	2	8	75·6	10	4	··	110	··	Negative	Positive	Negative	Yes	No	Possible	Yes**
5144	45	Male	6	15	1	353	37·7	14	3	70%	90	70%	Negative	Positive	Negative	Yes	No	Possible	Yes||
5238	14	Male	14	15	1	3	40·4	6	2	··	180	100	Negative	Positive	Negative	Yes	Yes	Possible	No
5116	39	Male	30	14	2	29	2·8	12	6	65%	440	30	Negative	Positive	Negative	Yes	No	Probable	No
5202	32	Male	7	14	2	85	50·5	9	15	55%	590	39	Negative	Positive	Negative	Yes	Yes	Probable	Yes
5245	25	Female	14	11	2	98	2·9	8	15	75%	540	47	Negative	Positive	Negative	No	Yes	Probable	No

All participants with tuberculous meningitis, listed by participant ID, were negative for cryptococcal antigen in blood and CSF. GCS=Glasgow Coma Scale. MRC=British Medical Research Council clinical stage of tuberculous meningitis. ART=antiretroviral therapy. CSF=cerebrospinal fluid. CRP=C-reactive protein. Xpert=Xpert MTB/RIF. ··=Missing data.

* CSF analysis.

† Percentage of CSF white cells that are lymphocytes; this was not always reported by the laboratory for CSF white cell count <5 cells per μL.

‡ Final physician diagnosis of tuberculous meningitis during hospitalisation (excluding Xpert Ultra results or delayed culture growth occurring after hospital discharge or death).

§ Participants were classified as definite, probable, possible, or not tuberculous meningitis on the basis of consensus uniform case definitions,^10^ excluding Xpert Ultra results.

¶ Grossly bloody; white cell count not done.

|| Known to have died after hospital discharge.

** Lost to follow-up after hospital discharge.

We assessed diagnostic performance for Xpert Ultra, Xpert, and culture compared with microbiologically proven, definite tuberculous meningitis positive by any of the three methods and with the uniform clinical case definition for definite or probable tuberculous meningitis (table 3). Xpert Ultra produced a sensitivity of 95% (21 of 22 positive cases) for microbiologically proven, definite tuberculous meningitis, which was superior to either Xpert (45%; ten of 22; p=0·0010) or culture (45%; ten of 22; p=0·0034; table 3). The negative predictive value for a negative Xpert Ultra was 99% (106 of 107 negative cases) in excluding microbiologically proven tuberculous meningitis. Conversely, the negative predictive value was 90% (107 of 119 negative cases) for both Xpert and culture. The time to positive mycobacteria culture was a median of 16 days (IQR 14–23). Among ten people with positive CSF cultures, seven died before cultures grew, with the median time to culture positivity being 17 days (IQR 13–21, range 9–43) after death. By comparison, the Xpert Ultra assay run time was 83 min.

**Table 3:** CSF diagnostic performance for tuberculous meningitis of Xpert, culture, and Xpert Ultra

	**Sensitivity *vs* composite endpoint (95% CI; n/N)**	**Sensitivity *vs* case definition (95% CI; n/N)**	**Assay error rate**
Xpert Ultra	95% (77–99; 21/22)	70% (47–87; 16/23)	2·3% (3/129)
Xpert	45% (24–68; 10/22)	43% (23–66; 10/23)	4·7% (6/129)
MGIT culture	45% (24–68; 10/22)	43% (23–66; 10/23)	1·6% (2/129)

All three tests were done in all 129 participants. Composite endpoint included any positive CSF Xpert Ultra, Xpert, or Bactec960 MGIT culture. Sensitivity *vs* uniform clinical case definition for definite (n=14) or probable (n=9) tuberculous meningitis excluded Xpert Ultra results in defining case status.^10^ Error in culture reflects contamination with non-tuberculous mycobacterium growth. Xpert=Xpert MTB/RIF. MGIT=mycobacteria growth indicator tube. CSF=cerebrospinal fluid.

Next-generation sequencing was done on seven Xpert Ultra positive cartridges (selected owing to availability). Six cartridges had a sufficient residual volume present inside the cartridge to successfully extract DNA and identify *M tuberculosis* genes by next-generation sequencing (table 4). Of these six cartridges, two had been only positive by Xpert Ultra (table 4). The seventh cartridge had a residual volume of less than 5 μL present after storage, resulting in unsuccessful sequencing (table 4).

**Table 4:** Next-generation sequencing results of *Mycobacterium tuberculosis* DNA

	**Xpert Ultra category**	**MGIT culture**	**Xpert**	**Next-generation sequencing of *M tuberculosis genes***
5001	Trace	Negative	Negative	IS6110 detected
5004	Very low	Positive	Positive	IS6110, IS1081, rpoB (WT) detected
5005	Trace	Positive	Negative	Negative*
5103	Trace	Positive	Negative	IS6110 detected
5194	Very low	Positive	Positive	IS6110, IS1081, rpoB (WT) detected
5116	Trace	Negative	Negative	IS6110 detected
5285	Very low	Negative	Positive	IS6110, IS1081, rpoB (WT) detected

Data presented for the seven participants, listed by participant ID, whose cartridges were available for testing. All other cartridges were promptly discarded per standard infection control procedures. IS6110 and IS1081 are insertion elements found exclusively with *M tuberculosis* complex. rpoB is the β subunit of RNA polymerase gene. Xpert=Xpert MTB/RIF. MGIT=mycobacteria growth indicator tube. WT=wild type without rifampin resistance.

* <5 μL available for testing.

We also classifed participants on the basis of the consensus uniform case definitions for tuberculous meningitis (appendix pp 2–5).^10^ When excluding Xpert Ultra results in the classification of definite tuberculous meningitis (to avoid incorporation bias), we diagnosed only 14 participants with definite tuberculous meningitis, and classified nine as probable (ie, total diagnostic score of ≥10, with ≥2 points coming from CSF criteria). We classified 56 participants as having possible tuberculous meningitis (total diagnostic score of 6–9 points). 50 participants were classified as not having tuberculous meningitis as defined by a known alternative diagnosis of cryptococcal meningitis (n=16) or *Streptococcus pneumoniae* meningitis (n=2); or with no convincing evidence of tuberculous meningitis (eg, score <6 points, no alternative diagnosis; n=32).

We compared Xpert Ultra testing of CSF to the uniform clinical case definition categories of probable or definite tuberculous meningitis wherein Xpert Ultra results were excluded (table 3). Culture and Xpert shared the same sensitivity for probable or definite tuberculous meningitis (table 3). For Xpert Ultra, we found higher sensitivity compared with Xpert or culture (table 3) and a negative predictive value of 93% (100 of 107 negative cases). If one assumed Xpert Ultra detection of *M tuberculosis* DNA in CSF represents false positivity in a person living with AIDS (which we believe is improbable), the diagnostic specificity was 95% (101 of 106 negative cases) among possible or non-tuberculous meningitis. Of these five putative Xpert Ultra false positives, four died and one was lost to follow-up (presumed dead). However, we believe these are not false positives. Among the three other participants with probable tuberculous meningitis that were positive by Ultra only, two participants received empirical therapy, one of whom survived (table 2).

Xpert Ultra results include semi-quantitative categories of trace, very low, low, moderate, and high. Of 21 Xpert Ultra positive specimens, nine (43%) were trace, seven (33%) were very low, and five (24%) were low (table 2). Of the eight samples that were positive only by Xpert Ultra, six (75%) were trace and two (25%) were very low. Overall, the median CD4 of participants according to category was 36 cells per μL (IQR 16–61) for trace, 85 cells per μL (72–133) for very low, and 73 cells per μL (61–124) for low.

Rifampin resistance by Xpert Ultra testing was not detected in 13 cases, and nine cases were indeterminate, all of which had trace quantification. No discordance occurred with resistance testing by Xpert or culture.

CSF collection volume remained important. Among participants without cryptococcal meningitis (who require large volume lumbar punctures for control of intracranial pressure), testing of at least 6 mL of CSF (median 10 mL [IQR 8–12]) was associated with a tuberculous meningitis diagnosis (19 [26%] of 73) compared with less than 6 mL (4 mL [3–5mL]; 3 [7%] of 43; p=0·014)].

## Discussion

In this HIV-infected cohort, Xpert Ultra testing demonstrated greater sensitivity when compared with culture or Xpert for tuberculous meningitis diagnosis. Xpert Ultra showed 95% sensitivity compared with 45% sensitivity for Xpert or MGIT culture. Importantly, of the 21 cases detected by Xpert Ultra, eight (38%) were not detected by Xpert or MGIT culture. One person was detected only by culture. Of the eight cases detected only by Ultra, case definitions categorised three as probable tuberculous meningitis, three as possible, and two as non-tuberculous meningitis. Importantly, most patients did not have imaging outside of the CNS completed and none had CNS imaging completed. Imaging might have pushed the two non-tuberculous cases into the possible tuberculous meningitis category, but also reflect the possible limitations of a clinical case definition. Alternatively, these tuberculous meningitis cases might be of a different phenotype from that which is typically detected or—although less likely—the results might be falsely positive. Although the examination of CSF specimens by Xpert Ultra was from cryopreserved samples, this study represents a potentially large step forward in the diagnosis of tuberculous meningitis.

A diagnostic test exhibiting greater than 90% sensitivity for tuberculous meningitis with results available in less than 90 min holds great potential to improve patient outcomes, particularly in detecting those with the lowest burden of infection. Further, a major concern in using the original Xpert for diagnosing tuberculous meningitis was an inadequate negative predictive value to rule out tuberculous meningitis.^13^, ^14^ The improved negative predictive value found with Xpert Ultra would constitute a major improvement. However, the negative predictive value was 99% against definite tuberculous meningitis but was only 93% against probable or definite tuberculous meningitis. Thus, clinical judgment remains paramount, and for this Xpert Ultra cannot substitute.

Tuberculous meningitis is a paucibacillary disease.^20^ Thus, any diagnostic assay seeking to microbiologically detect tuberculous meningitis must optimise the limit of detection while maintaining specificity. MGIT culture has an analytical sensitivity threshold of about ten bacilli (colony-forming units [CFU] per mL) whereas Xpert has a threshold of about 100 CFU/mL.^9^, ^21^ Xpert Ultra has a limit of detection of about 10 CFU/mL,^15^ which probably explains the improved sensitivity of Xpert Ultra as compared with Xpert. Re-engineering of the Ultra cartridge allows double the volume of DNA to reach the PCR reaction. However, the volumes of centrifuged CSF available for this study (1·0 mL for Xpert and 0·5 mL for Xpert Ultra) actually put the Xpert Ultra at a disadvantage, making its performance all the more impressive. The added value of cartridge re-engineering is likely to be particularly important in paediatric tuberculous meningitis, in which volume of CSF is often small.

The Xpert Ultra semi-quantitative results also provide evidence of the lower analytical threshold of detection playing a role in the assay's improved performance. Nearly half (nine of 21) of samples detected by Xpert Ultra were only detected in trace amounts. Of those nine samples, only one was positive by Xpert and only two by culture.

Xpert Ultra also showed improved sensitivity compared with culture despite similar analytic detection thresholds between the two modalities. This difference is most likely due to non-viable bacilli being detected by PCR but not by culture. In this study, two of 22 participants with definite tuberculous meningitis were already on pulmonary tuberculous therapy, which probably contributed to the presence of non-viable bacilli. Two participants had reportedly completed pulmonary tuberculous therapy in the past year but had developed new meningitis after starting HIV therapy. In the one negative Xpert Ultra participant, sample-to-sample variation might have resulted in a positive culture and negative Xpert Ultra. Centrifugation of samples (>6 mL) and testing a larger CSF volume (1–2 mL) with the Xpert Ultra cartridge might mitigate this possibility.

Importantly, in the field validation trial of Xpert Ultra for pulmonary tuberculosis, investigators found 95·6% specificity for the Xpert Ultra and 98·3% specificity for Xpert overall.^16^ This finding was felt to be largely due to detection of previously treated antecedent tuberculous infection, where, among these patients, the specificity was 93·2% for Xpert Ultra and 98·0% for Xpert. Given that for tuberculous meningitis, either the mycobacteria are cleared or the disease progresses to cause death, this cause of lower specificity in pulmonary tuberculous should not apply to CSF. Indeed, tuberculous bacilli found in the CSF are very unlikely to be due to past disease and almost certainly represent tuberculous meningitis in this immunocompromised population. Additional molecular testing for 14 common meningitis aetiologies did not reveal any other explanatory pathogens in CSF positive for Xpert Ultra only.^18^ Furthermore, next-generation sequencing was able to independently confirm *M tuberculosis* DNA when extracted from six stored Ultra-positive cartridges.

Because the study population were all HIV-infected, the findings might not be generalisable to people without HIV infection. Study limitations include the absence of a true gold standard for tuberculous meningitis diagnosis and absence of post-mortem examinations.^10^ In using a composite reference standard, incorporation bias is a potential concern. Yet, even when excluding the Xpert Ultra results, Xpert Ultra had the highest sensitivity for definite or probable tuberculous meningitis using the consensus clinical case definition.^10^ Although the Xpert Ultra sensitivity was only 70% compared with a case definition of probable or definite tuberculous meningitis, the case definition is probably imperfect but does provide cross-study comparability. Despite these limitations and the relatively modest number of tuberculous meningitis cases, we believe the principles in this study can be extrapolated. Xpert Ultra should be a first-line test for tuberculous meningitis after excluding cryptococcal infection.

In summary, we tested the performance of Xpert Ultra for tuberculous meningitis detection on centrifuged CSF. Xpert Ultra showed clearly improved sensitivity compared with either Xpert or MGIT culture. Xpert Ultra's combination of lower analytic limit of detection and ability to detect dead bacilli after starting tuberculosis or HIV therapy vastly improves sensitivity compared with either culture or Xpert. Prospective Xpert Ultra testing for detection of tuberculous meningitis in a larger population is needed to confirm these findings.
