# Supplementary material for: Diagnostic accuracy of Xpert MTB/RIF Ultra for tuberculous meningitis in HIV-infected adults: a prospective cohort study
Source: Lancet Infect Dis. 2018 Jan;18(1):68–75. doi: 10.1016/S1473-3099(17)30474-7 (PMC5739874; doi:10.1016/S1473-3099(17)30474-7)
Supplement: Supplementary appendix [file mmc1.pdf]

THE LANCET Infectious Diseases

Supplementary webappendix

This webappendix formed part of the original submission and has been peer reviewed. We post it as supplied by the authors.

Supplement to: Bahr NC, Nuwagira E, Evans EE, et al, on behalf of the ASTRO-CM Trial Team. Diagnostic accuracy of Xpert MTB/RIF Ultra for tuberculous meningitis in HIV-infected adults: a prospective cohort study. *Lancet Infect Dis* 2017; published online September 14. http://dx.doi.org/10.1016/S1473-3099(17)30474-7.

Supplementary Appendix:

This appendix has been provided by the authors to give readers additional information about their work.

ASTRO-CM Trial Team members include: Darlisha A Williams, Kabanda Taseera, Dan Nyehangane, Mugisha Ivan, Patrick Orikiriza, Joshua Rhein, Kathy Huppler Hullsiek, Abdu Musubire, Katelyn Pastick, James Mwesigye, Pamela Nabeta (FIND).

Appendix Contents

Tuberculous meningitis: a uniform case definition for use in clinical research [10] 2
Performance of Uniform Clinical Case Criteria 4
Next Generation DNA Sequencing 5
Appendix Table S1: Xpert MTB/Rif Ultra Semi-Quantitative Category 6
Appendix Figure S1: Schematic of CSF processing after collection 7

Tuberculous meningitis: a uniform case definition for use in clinical research [10]

Definite tuberculous meningitis

- Symptoms and signs of meningitis
  AND
- Definitive diagnosis by:
  - CSF acid-fast bacilli
  - CSF *Mycobacterium tuberculosis* culture
  - CSF positive commercial nucleic acid amplification test.
  - Acid-fast bacilli seen in the context of histological changes consistent with tuberculosis in the brain or spinal cord (i.e. on autopsy).

Probable tuberculous meningitis

- Symptoms and signs of meningitis, plus:
- Total diagnostic score of:
  - ≥10 points when cerebral imaging is not available or
  - ≥12 points when cerebral imaging is available plus
- At least 2 points should either come from CSF or cerebral imaging criteria.
- Exclusion of alternative diagnoses.

Possible tuberculous meningitis

- Symptoms and signs of meningitis, plus:
- Total diagnostic score of:
  - 6-9 points when cerebral imaging is not available or
  - 6-11 points when cerebral imaging is available plus
- Exclusion of alternative diagnoses.
- Cannot be diagnosed or excluded without a lumbar puncture or cerebral imaging.

Not tuberculous meningitis

- Alternative diagnosis established, without a definitive diagnosis of tuberculous

This uniform case definition for TB meningitis is meant for use in clinical research to standardize reporting of across different research studies.

The uniform case definition is not meant as the sole diagnostic criteria for clinical practice and should not supplant expert clinical judgement.

Tuberculous meningitis: a uniform case definition diagnostic scoring table

Diagnostic criteria for classification of definite, probable, possible, and not TB meningitis

**Clinical criteria**	(Max category score=6)
Symptom duration of >5 days	4
Systemic symptoms suggestive of tuberculosis, e.g. weight loss (or poor weight gain in children), night sweats, or persistent cough for >2 weeks	2
Pediatrics <10 years old: History of recent within past year close contact with an individual with pulmonary tuberculosis or a positive tuberculin skin test or interferon-gamma release assay	2
Focal neurological deficit, excluding cranial nerve palsies	1
Cranial nerve palsy	1
Altered consciousness	1
**CSF criteria**	(Max category score=4)
Clear appearance	1
White Cells: 10–500 per μL	1
Lymphocytic predominance (>50%)	1
Protein concentration >100 mg/dL (1 g/L)	1
CSF to plasma glucose ratio of <50% or CSF glucose <40 mg/dL (2.2mmol/L)	1
**Cerebral Imaging criteria***	(Max category score=6)
Hydrocephalus	1
Basal meningeal enhancement	2
Tuberculoma	2
Infarction	1
Pre-contrast basal hyperdensity	2
**Evidence of Tuberculosis Elsewhere**	(Max category score=4)
Chest radiograph suggestive of active TB = 2; suggestive of miliary TB=4	2/4
CT/ MRI/ ultrasound evidence for tuberculosis outside the CNS*	2
AFB identified or *Mycobacterium tuberculosis* cultured from another non-CNS source, e.g. sputum, lymph node, gastric washing, urine, blood culture	4
Positive commercial *M tuberculosis* nucleic acid amplification test (e.g. PCR) from non-CNS specimen	4
**Exclusion of alternative diagnoses** †	

*Working CT was not available at Mbarara Regional Referral Hospital in 2015-2016.

† Exclusionary CSF diagnostics included: cryptococcal antigen lateral flow assay (Immy, Norman, Oklahoma), Gram's Stain, bacterial culture, FilmArray Meningitis/Encephalitis PCR (Biofire Diagnostics, Salt Lake City, Utah).

Performance of Uniform Clinical Case Criteria

Of the 22 definite TBM cases by composite definition of any positive CSF test, in the absence of the definitive microbiologic testing, 5 (23%) persons had probable TBM (score ≥10), 14 (64%) possible TBM (score 6-9), and 3 (14%) with non-TBM (score <6). None of the definitive TBM had an alternative diagnosis, and all were cryptococcal antigen negative in blood and in CSF. All CSF cultures were negative for aerobic bacteria and *Cryptococcus*. Among the eight Xpert Ultra positive only specimens, without Xpert Ultra results, the categorizations were three as probable TBM, three as possible TBM, and two as non-TBM category.

Among 107 persons without definitive, microbiologic-proven TBM, 6 (5.6%) had probable TBM, 53 (50%) possible TBM, and 48 (45%) non-TBM in the absence of additional microbiologic testing. Of the 48 with non-TBM, confirmed alternative etiologies included *Cryptococcus neoformans* (n=16) and *Streptococcus pneumoniae* (n=2). Among the 16 persons with cryptococcal meningitis, in the absence of cryptococcal antigen testing, categorization of probable TBM would have occurred for 2, possible TBM for 11, and non-TBM for 3.

Among the eight Xpert MTB/RIF Ultra positive only specimens, these CSF were also run on the FDA-approved FilmArray® meningitis/encephalitis multiplex PCR panel. This panel tests for: *Streptococcus pneumoniae*, *Neisseria meningitidis*, *Haemophilus influenzae*, *Listeria monocytogenes*, *Streptococcus agalactiae*, *Escherichia coli*, cytomegalovirus (CMV), enterovirus, herpes simplex virus 1 (HSV-1), herpes simplex virus 2 (HSV-2), human herpes virus 6 (HHV-6), human parechovirus, varicella zoster virus (VZV), and *Cryptococcus neoformans/gattii*. All Xpert Ultra positive specimens were negative by FilmArray® testing.

There were no adverse events related to the performance of any of the diagnostic tests.

The Xpert Ultra sensitivity versus the clinical case definition of 70% (16/23) has a 95% confidence interval of 47% to 87%. In considering the clinical case definition performance with incorporation of positive Xpert Ultra results into the total number of

cases observed, the sensitivity of the case definition was 82% (23/28) for probable/definite TB meningitis without Xpert Ultra results.

Next Generation DNA Sequencing

Seven positive Xpert Ultra cartridges were stored at -80^{0}C and then transported at -20^{0}C to the Emerging Bacteria Pathogen Unit at San Raffaele Scientific Institute in Milan, Italy. The other 14 positive Ultra cartridges had been discarded promptly as per standard infection control practices before the -80^{0}C storage could occur.

In seven Xpert Ultra cartridges, the residual volume of liquid, potentially containing DNA amplicons, were extracted from the Xpert ultra cartridges. Paired-end libraries with 2x150 read length were prepared using the Nextera XT DNA Sample Preparation kit (Illumina Inc., San Diego, CA, USA) and sequenced on an Illumina MiniSeq platform according to the manufacturer's instructions. The output fastq data files were assembled using Velvet as short read assembler, trimming reads at 5' and 3' end until average quality was ≥30 in a window of 20 bases. Sequence analysis was performed using a dedicated bioinformatics pipeline to identify *M. tuberculosis* gene sequences for: β subunit of RNA polymerase gene (rpoB); IS6110 and IS1081 insertion elements found exclusively with *M. tuberculosis* complex.

Of the seven cartridges sent for sequencing, upon arrival at the San Raffaele Scientific Institute, six cartridges had residual fluid able to be extracted. In all six, *M. tuberculosis* specific genes were identified, as presented in manuscript Table 3.

Appendix Table S1: Xpert MTB/Rif Ultra Semi-Quantitative Category

**Xpert Ultra Semi-quantitative Category**			Mycobacterial Culture		Total
			Negative	Positive	
**Negative**	Xpert MTB/Rif	Xpert Negative		1	1
		Xpert Positive			0
	Total			1	1
**Trace**	Xpert MTB/Rif	Xpert Negative	6	2	8
		Xpert Positive	1		1
	Total		7	2	9
**Very Low**	Xpert MTB/Rif	Xpert Negative	2	1	3
		Xpert Positive	2	2	4
	Total		4	3	7
**Low**	Xpert MTB/Rif	Xpert Negative			0
		Xpert Positive	1	4	5
	Total		1	4	5
**Total**	Xpert MTB/Rif	Xpert Negative	8	4	12
		Xpert Positive	4	6	10
	Total		12	10	22

Xpert Ultra results include semi-quantitative categories of: trace, very low, low, moderate, and high. Of 21 Xpert Ultra positive specimens, nine (43%) were ‘trace’, seven (33%) were ‘very low’, and five (24%) were ‘low.’ Of nine ‘trace’ Ultra specimens, one (11%) was Xpert positive and two (22%), culture positive. Of the seven categorized as ‘very low’ by Xpert Ultra, four (57%) were positive by Xpert and three (43%) by culture. Of the five samples categorized as ‘low’ by Xpert Ultra, five (100%) were positive by Xpert and four (80%) by culture. Of the eight samples that were positive only by Xpert Ultra, six (75%) were ‘trace’ and two (25%) were ‘very low.’

Appendix Figure S1: Schematic of CSF processing after collection

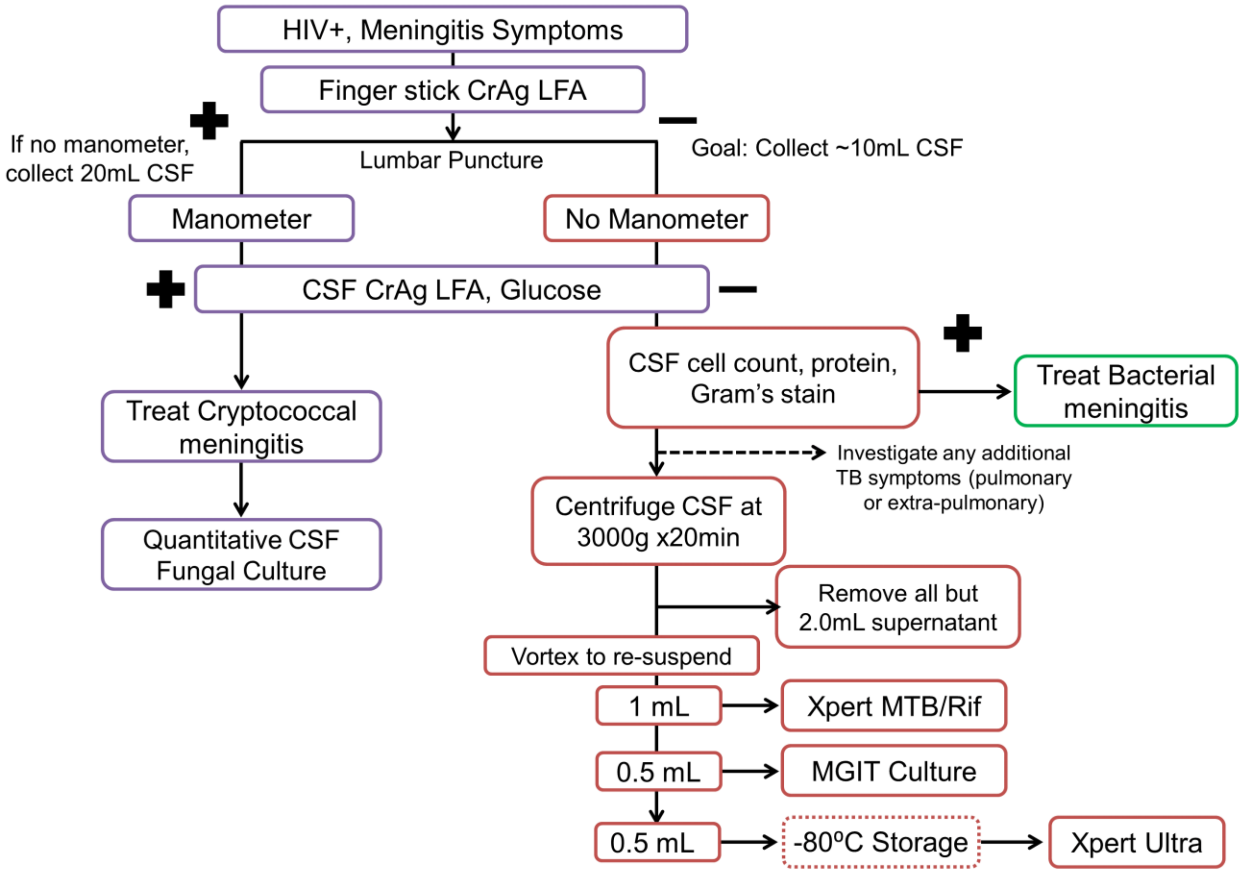

The figure shows the process by which CSF was handled after collection in order to accomplish Xpert MTB/Rif, Xpert MTB/Rif Ultra, and TB Mycobacteria growth indicator tube (MGIT) culture. Cryptococcal antigen (CrAg) testing was performed as the initial triage test by finger stick at the bedside. All persons who were CSF CrAg negative had a TB work up. CSF acid fast bacilli staining was not performed to maximize the testing volume for culture, Xpert, and Ultra, based on the principles of Durski et al to optimize CSF testing in a cost-effective manner.[1]
